# Supplementary material for: Dairy manure, glyphosate, and antimicrobials (copper, streptomycin, and triazole) modulated the composition of antimicrobial resistance at the gene and microbial levels in a processing tomato field
Source: Microbiol Spectr. 2026 Mar 17;14(4):e02003-25. doi: 10.1128/spectrum.02003-25 (PMC13055215; doi:10.1128/spectrum.02003-25)
Supplement: Supplemental figures — Figures S1, S2, and S4 to S10. [file spectrum.02003-25-s0001.pdf]

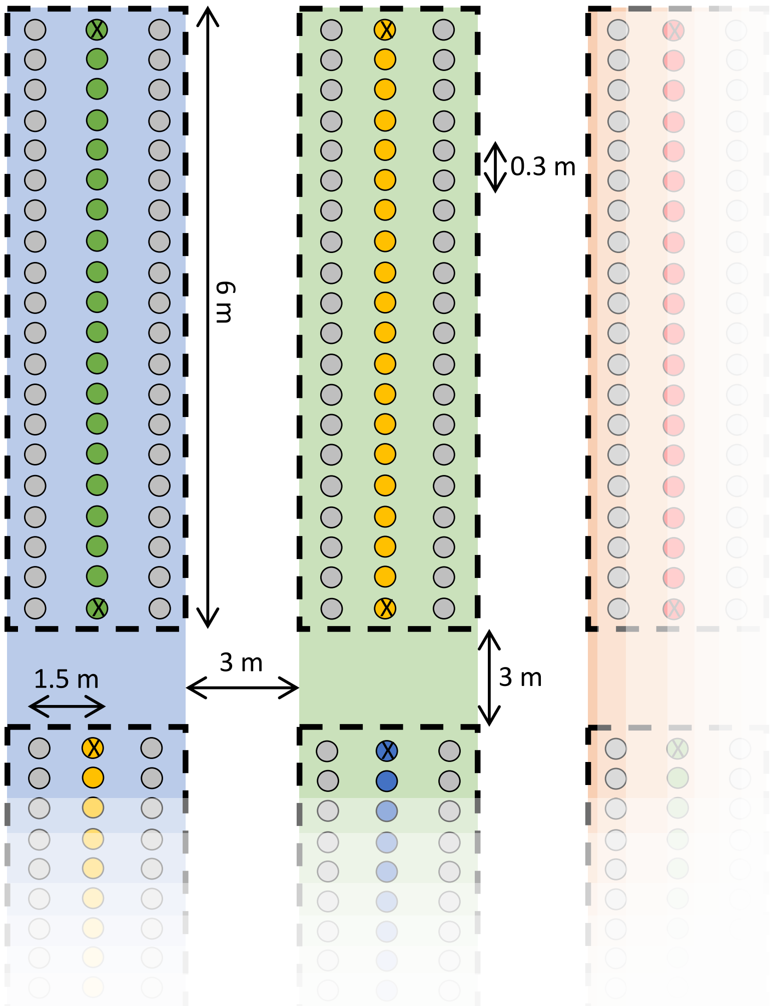

Supplemental figure 1. Field design. The field was composed of 16 treatment rows of 33.4-meter-long, separated by 3-meter-wide alleys covered with grass (white area between the rows). Each treatment row was composed of two border rows (gray rows) and one experimental row in the middle (colored row), and each separated by 1.5-meter-wide alleys with no cover crop. In addition, treatment rows were divided, in length, into four experimental plots of 6-meter-long (delimited by the dotted line). Experimental plots, within a treatment row, were separated with 3-meter-wide alleys with no cover crop. The color used for the treatment rows and the experimental plots illustrate the use of different health (for the rows) and disease (for the plots) management practices. The weekly application of the antimicrobial agents (copper, streptomycin, and propiconazole) was performed only on the experimental rows. One plot contained 20 plants per row (total of 60 plants per plot and 240 plants per row across the whole field selected for this study) . The plants located in the border of the plot (colored circles with an "X" and gray circles) were not selected for sampling of the soil and leaf tissues.

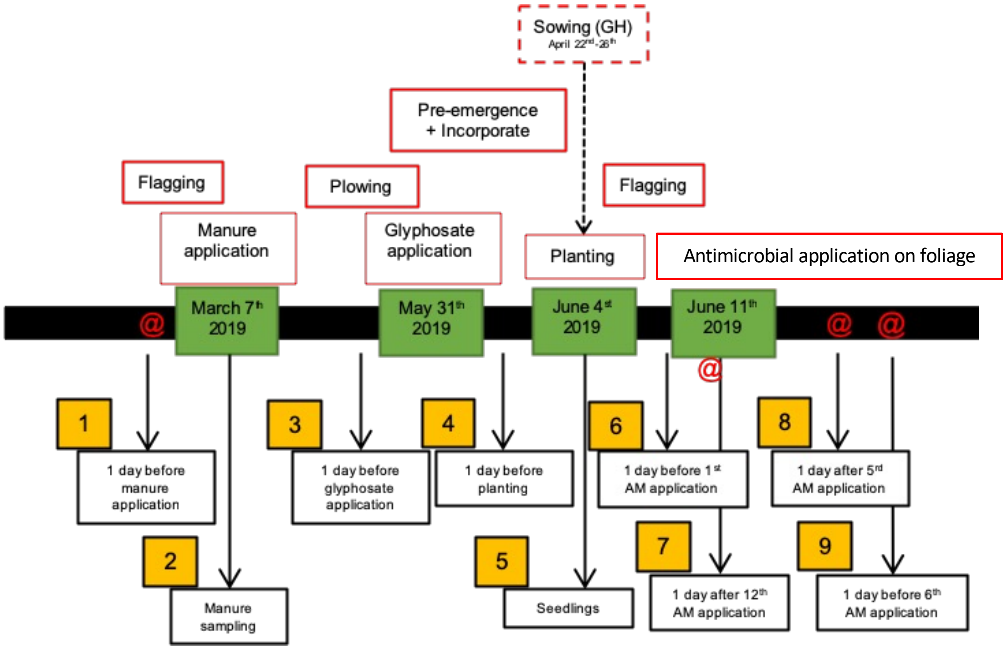

Supplemental Figure 2. Timeline for the health and disease management practices and sampling time points. Red/white box: field task; Green box: day of the field task; Black/white box: sampling details; Orange box: time point (TP) order. @: measurement of the soil properties. AM: antimicrobial. GH: Greenhouse (Selby Hall, CFAES, Wooster).

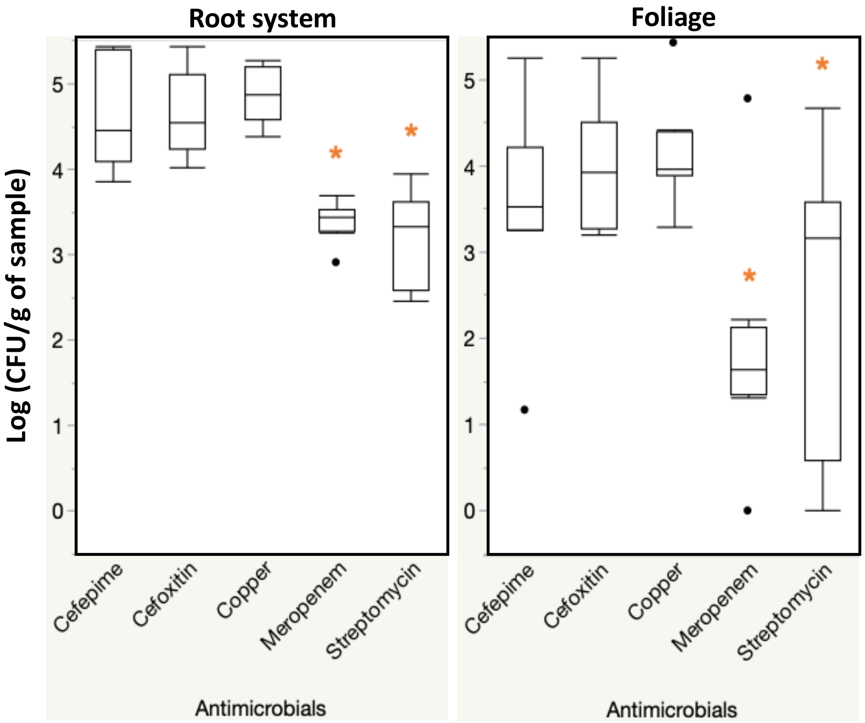

Supplemental Figure 4. Antimicrobial resistant bacteria from the seedlings (roots and foliage) collected before transplantation in the field (TP5). A) Quantification of culturable bacteria resistant to 200 µg/ml copper, 200 µg/ml streptomycin, 4 µg/ml cefepime, 8 µg/ml cefoxitin, and 1 µg/ml of meropenem by direct plating on MacConkey agar. *: bacterial populations resistant to meropenem or streptomycin were significantly lower compared to the ones resistant to cefepime, cefoxitin, or copper (P<0.05; n= 8 seedlings). The culturable bacteria resistant to copper were the most abundant in both the foliage and the root system (4.15 [3.63-4.66] and 4.89 [4.62-5.16] CFU/g of sample, respectively), followed by cefoxitin (3.95 [3.35-4.55] and 4.67 [4.25-5.09] CFU/g of sample, respectively), cefepime (3.54 [2.56-4.51] and 4.64 [4.10-5.19] CFU/g of sample, respectively), streptomycin (3.43 [2.89-3.86] and 3.05 [2.75-3.66] CFU/g of sample, respectively), and meropenem (2.13 [1.20-3.06] and 3.39 [3.20-3.59] CFU/g of sample, respectively). All the culturable AMR bacteria, except the ones resistant to streptomycin, were significantly higher in the root system compared to the foliage (P<0.05).

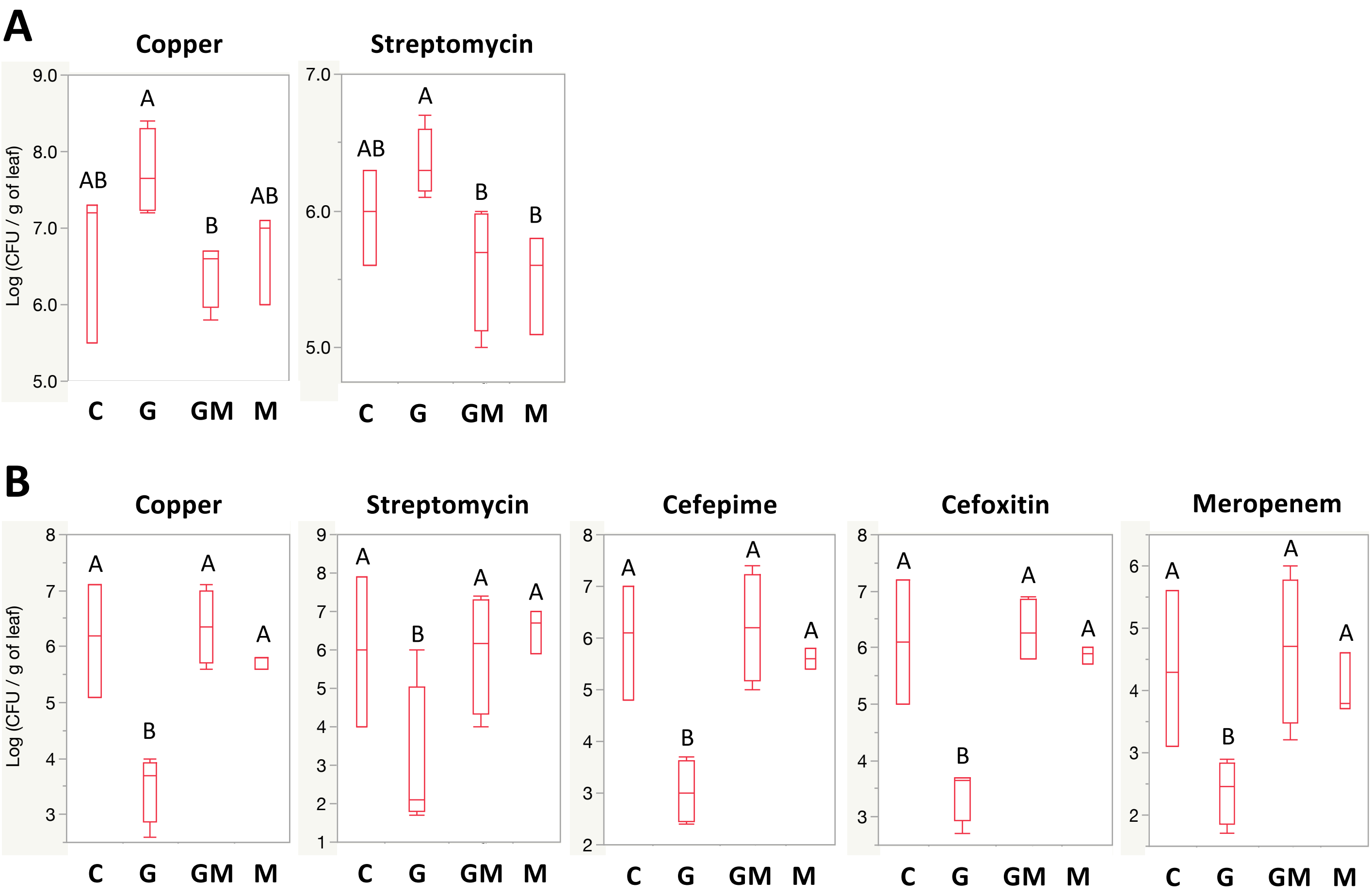

Supplemental Figure 5. Copper applications influenced the abundance of culturable antimicrobial resistant bacteria in the tomato leaf tissues collected from glyphosate applied rows at TP7 (A) and TP9 (B). No significant differences were detected between the health management practice groups at TP7 for the culturable bacteria resistant to cefepime, cefoxitin, and meropenem. Quantification of culturable bacteria resistant to 200 µg/ml copper, 200 µg/ml streptomycin, 4 µg/ml cefepime, 8 µg/ml cefoxitin, and 1 µg/ml of meropenem by direct plating on Mac Conkey agar. Letters represent statistical categories ($P<0.05$). C: non-treated rows; G, M, and GM: rows applied with manure and/or glyphosate. N= 4 pooled leaf samples per group.

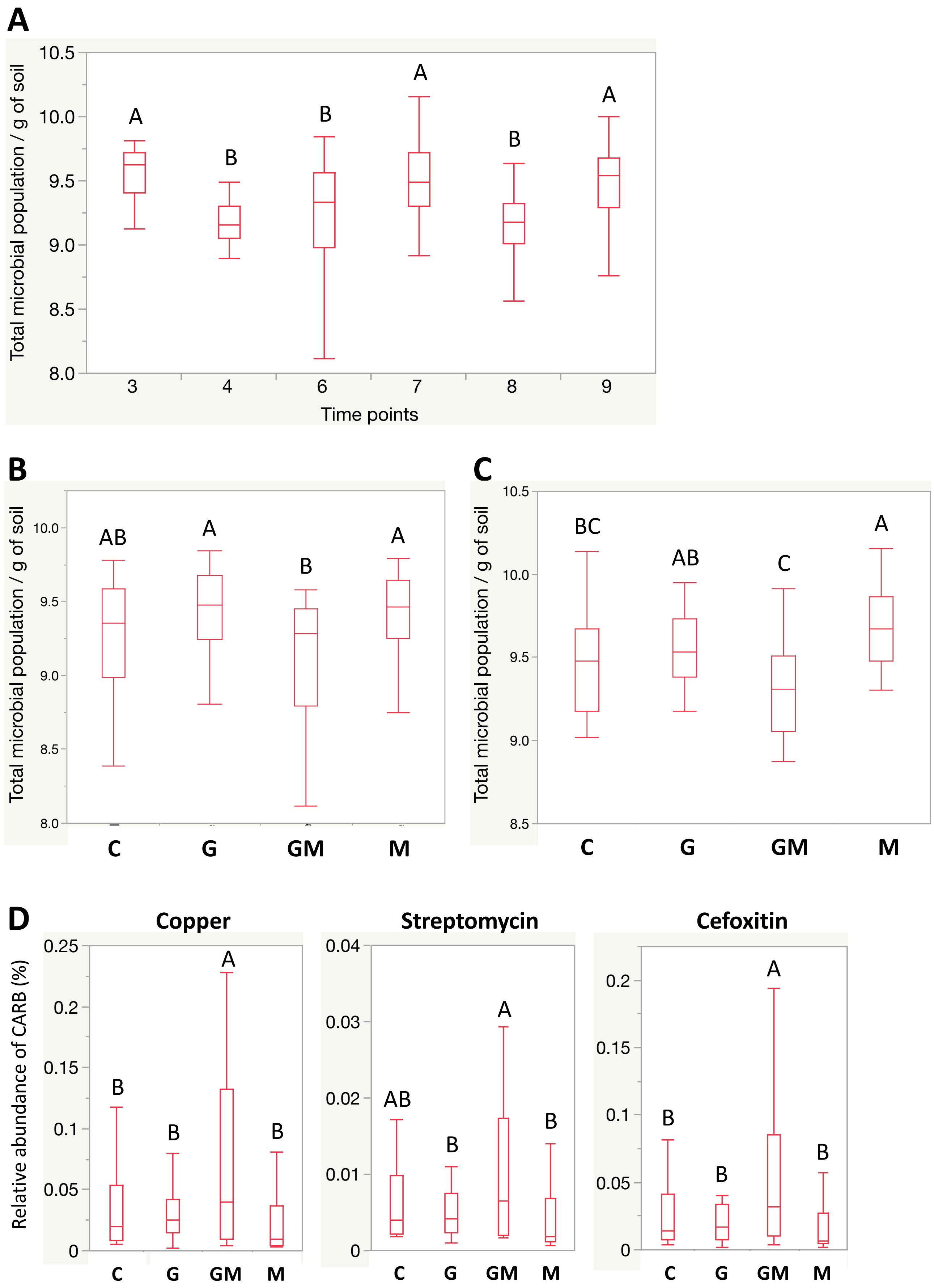

A
Total microbial population / g of soil
10.5
10.0
9.5
9.0
8.5
8.0
A
B
B
A
B
A
3
4
6
7
8
9
Time points
B
Total microbial population / g of soil
10.0
9.5
9.0
8.5
8.0
AB
A
B
A
C
G
GM
M
C
Total microbial population / g of soil
10.5
10.0
9.5
9.0
8.5
BC
AB
C
A
C
G
GM
M
D
Relative abundance of CARB (%)
Copper
0.25
0.2
0.15
0.1
0.05
0
B
B
A
B
C
G
GM
M
Streptomycin
0.04
0.03
0.02
0.01
0
AB
B
A
B
C
G
GM
M
Cefoxitin
0.2
0.15
0.1
0.05
0
B
B
A
B
C
G
GM
M

Supplemental Figure 6. Impact of manure and glyphosate application of the total micro-organism and CARB population of the soil at TP6 and TP7. A) Total microbial population (cell size <40 μm) of the soil overtime. The combination of manure plus glyphosate reduced the total microorganism population in the soil samples collected at TP6 (B) and TP7 (C). The total micro-organism detected in the soil samples was estimated using flow cytometry after staining the nucleic acid with SYBR green. D) Relative abundance of specific AMR bacteria (200 μg/ml copper, 200 μg/ml streptomycin, and 8 μg/ml cefoxitin) in the soil at TP7. C: non-treated rows; G, M, and GM: rows applied with manure and/or glyphosate. N= 16 pooled soil samples per group. Letters represent statistical categories ($P<0.05$). CARB: culturable antimicrobial resistant bacteria. AMR: antimicrobial resistance

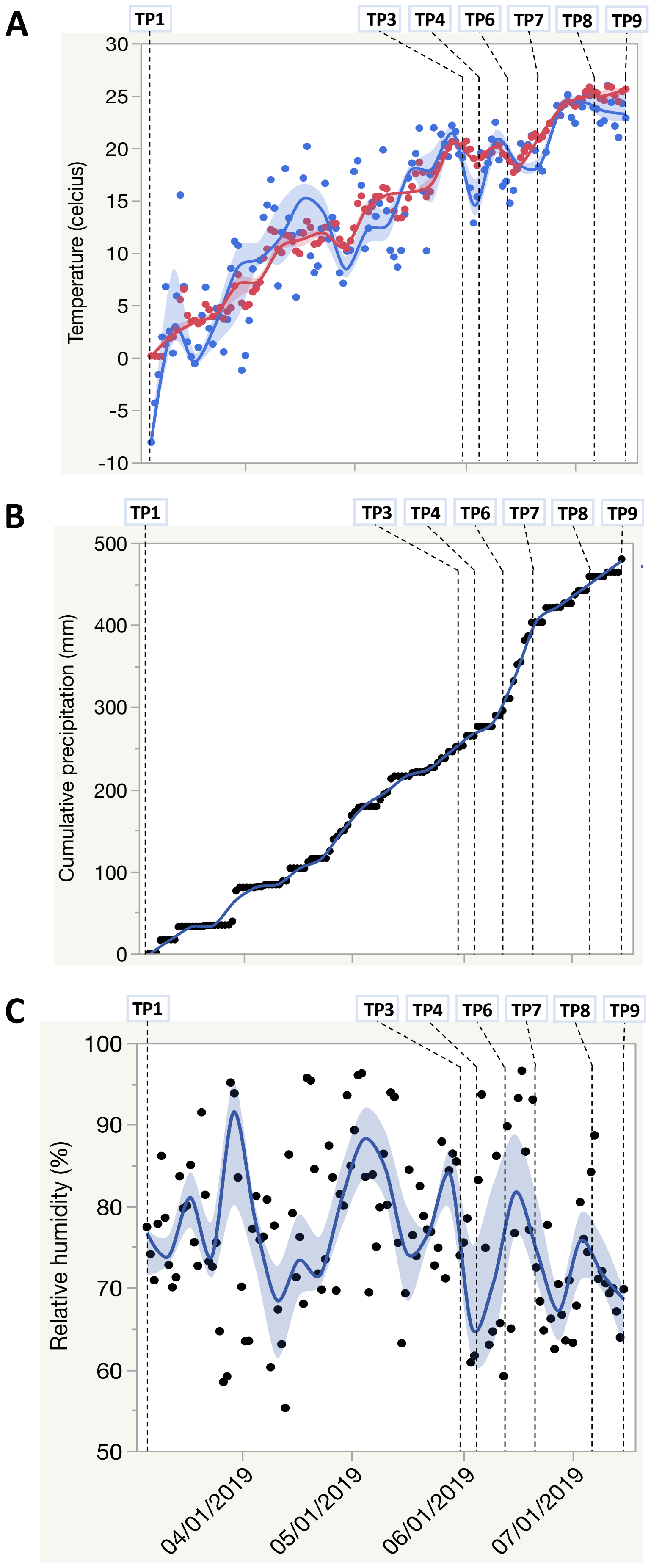
A
TP1
TP3
TP4
TP6
TP7
TP8
TP9
Temperature (celcius)
30
25
20
15
10
5
0
-5
-10
B
Cumulative precipitation (mm)
500
400
300
200
100
0
C
Relative humidity (%)
100
90
80
70
60
50
04/01/2019
05/01/2019
06/01/2019
07/01/2019

Supplemental Figure 7. Environmental data recorded between TP1 and TP9. A) Average temperatures of the air (blue line) and 5 cm deep in the soil (red line). B) Cumulative precipitation results. C) Average relative humidity data. Data were recorded hourly and displayed in each graph as an average or cumulative sum for each day. A smooth average (solid line) plus confidence if fit (shadow associated with the solid line) was generated for each data set. The dotted lines represent the soil and leaf sample collections' days (from TP1 to TP9). The data were extracted by the Ohio Agricultural Research Development Center, Wooster weather station (https://www.oardc.ohio-state.edu/weather1/stationinfo.asp?id=1). Overall, the environmental temperature influenced the abundance of specific culturable AMR detected in both the soil and leaf samples collected between TP1 and TP9. More precisely, the cumulative temperatures recorded 5 cm deep in the soil were positively correlated with the total abundance culturable bacteria, especially the ones resistant to cefepime (r^2= 0.25) and streptomycin (r^2= 0.69; $P<0.0001$). Only culturable bacteria resistant to meropenem showed no correction with the soil temperature. On the other hand, the cumulative temperature of the air was negatively correlated with the abundance of total abundance culturable bacteria, especially the ones resistant to copper (r^2= 0.20) and meropenem (r^2= 0.50; $P<0.0001$). No correlation between the abundance culturable bacteria and the relative humidity was detected. Further, the abundance of each culturable AMR bacteria were highly correlated (r^2> 0.70) between each other in both the soil and leaf samples collected between TP1 and TP9, except with the streptomycin resistant bacteria ($0.31 < r^2 < 0.58$).

A

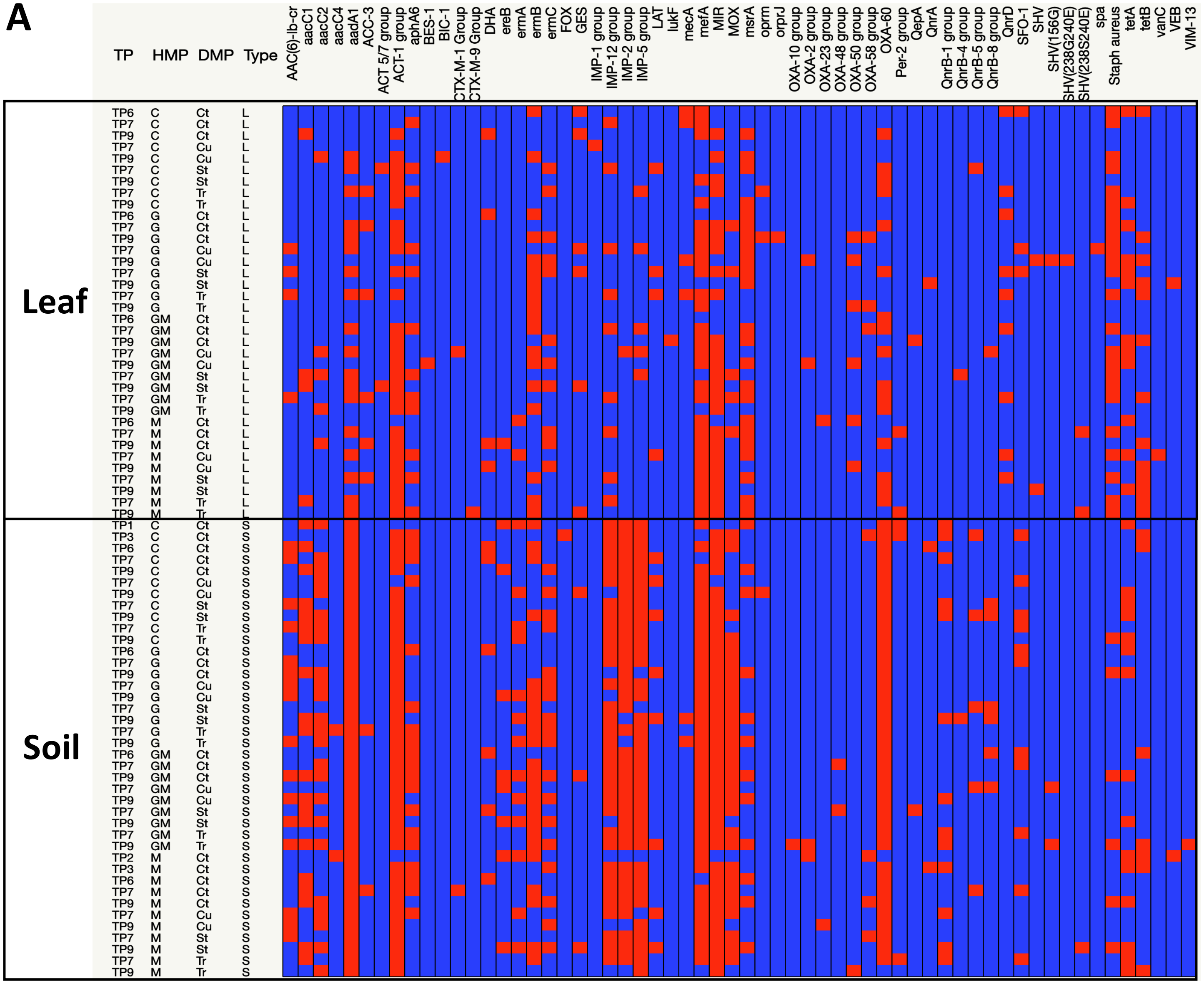
TP
HMP
DMP
Type
AAC(6)-Ib-cr
aacC1
aacC2
aacC4
aadA1
ACC-3
ACT 5/7 group
ACT-1 group
aphA6
BES-1
BIC-1
CTX-M-1 Group
CTX-M-9 Group
DHA
ereB
ermA
ermB
ermC
FOX
GES
IMP-1 group
IMP-12 group
IMP-2 group
IMP-5 group
LAT
lukF
mecA
mefA
MIR
MOX
msrA
oprm
oprJ
OXA-10 group
OXA-2 group
OXA-23 group
OXA-48 group
OXA-50 group
OXA-58 group
OXA-60
Per-2 group
QepA
QnrA
QnrB-1 group
QnrB-4 group
QnrB-5 group
QnrB-8 group
QnrD
SFO-1
SHV
SHV(156G)
SHV(238G240E)
SHV(238S240E)
spa
Staph aureus
tetA
tetB
vanC
VEB
VIM-13
Leaf
TP6 C Ct L
TP7 C Ct L
TP9 C Ct L
TP7 C Cu L
TP9 C Cu L
TP7 C St L
TP9 C St L
TP7 C Tr L
TP9 C Tr L
TP6 G Ct L
TP7 G Ct L
TP9 G Ct L
TP7 G Cu L
TP9 G Cu L
TP7 G St L
TP9 G St L
TP7 G Tr L
TP9 G Tr L
TP6 GM Ct L
TP7 GM Ct L
TP9 GM Ct L
TP7 GM Cu L
TP9 GM Cu L
TP7 GM St L
TP9 GM St L
TP7 GM Tr L
TP9 GM Tr L
TP6 M Ct L
TP7 M Ct L
TP9 M Ct L
TP7 M Cu L
TP9 M Cu L
TP7 M St L
TP9 M St L
TP7 M Tr L
TP9 M Tr L
Soil
TP1 C Ct S
TP3 C Ct S
TP6 C Ct S
TP7 C Ct S
TP9 C Ct S
TP7 C Cu S
TP9 C Cu S
TP7 C St S
TP9 C St S
TP7 C Tr S
TP9 C Tr S
TP6 G Ct S
TP7 G Ct S
TP9 G Ct S
TP7 G Cu S
TP9 G Cu S
TP7 G St S
TP9 G St S
TP7 G Tr S
TP9 G Tr S
TP6 GM Ct S
TP7 GM Ct S
TP9 GM Ct S
TP7 GM Cu S
TP9 GM Cu S
TP7 GM St S
TP9 GM St S
TP7 GM Tr S
TP9 GM Tr S
TP2 M Ct S
TP3 M Ct S
TP6 M Ct S
TP7 M Ct S
TP9 M Ct S
TP7 M Cu S
TP9 M Cu S
TP7 M St S
TP9 M St S
TP7 M Tr S
TP9 M Tr S

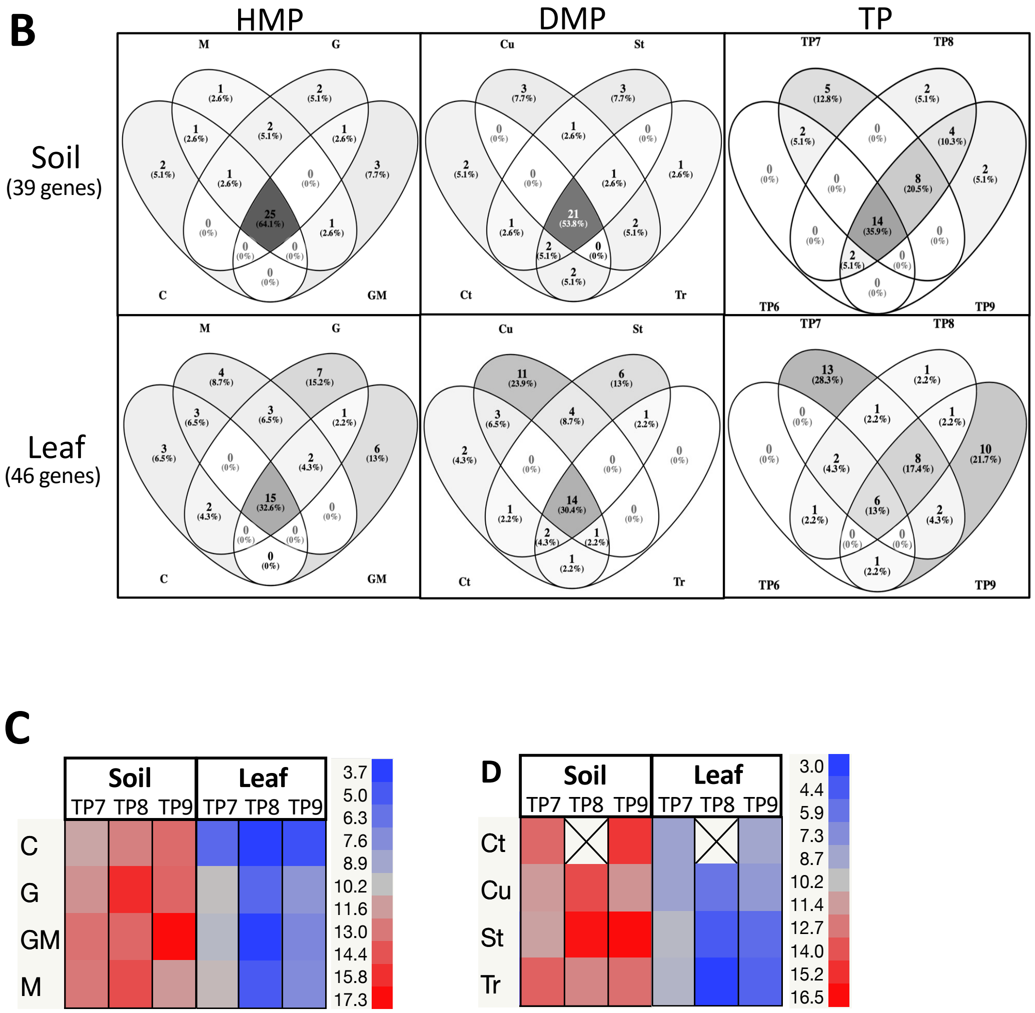

B
HMP
DMP
TP
Soil
(39 genes)
Leaf
(46 genes)
M
G
C
GM
Cu
St
Ct
Tr
TP7
TP8
TP6
TP9
C
Soil
Leaf
TP7 TP8 TP9 TP7 TP8 TP9
C
G
GM
M
3.7
5.0
6.3
7.6
8.9
10.2
11.6
13.0
14.4
15.8
17.3
D
Soil
Leaf
TP7 TP8 TP9 TP7 TP8 TP9
Ct
Cu
St
Tr
3.0
4.4
5.9
7.3
8.7
10.2
11.4
12.7
14.0
15.2
16.5

Supplemental Figure 8. Impact of HMP and DMP on the ARGs composition in leaf and soil samples between TP1 and TP9. A) ARG profile of the soil and leaf samples collected between TP1-TP9. The color of the cells is proportional to the abundance of a designated gene (inv.Ct values) in a given sample (labeled by sample type [leaf or soil]; health management practice [HMP]; disease management practice [DMP]; Time point [TP]). B) Venn diagram of the ARG diversity by HMP, DMP, or time points between TP6 and TP9. The color of the cells is proportional to diversity of ARGs. C) Total number of ARGs per samples by HMP or DMP between TP6 and TP9. The color of the cells is proportional to the number of total ARGs. TP1: 1 day before the beginning of the experiment; TP2: pure manure collected before its application in the field; TP3: 1 day before the glyphosate application ; TP6: 1 day before the first antimicrobial applications; TP7, TP8, and TP9: 6 days after the first, fourth, ad fifth antimicrobial applications, respectively. M, G, G+M: rows treated once with manure and/or glyphosate. Cu, St, and Tr: experimental plots treated weekly with copper, streptomycin, or triazole (propiconazole); C and Ct: controls (not treated). Genes associated with aminoglycoside (n=4/5; *aacC1*, *aacC2*, *aadA1*, and *aphA6*), beta-lactamase (class B [n=3/9; IMP-2, IMP-5, and IMP-12] and C [n=5/11; ACT-1, DHA, LAT, MIR, and MOX]), erythromycin (n=1/1; *ereB*), fluoroquinolone (n=3/11; AAC(6)-lb-cr, QnrB-1, and QnrB-5), and macrolide (n=4/5; *ermA*, *ermB*, *ermC*, and *mefA*) resistances were detected in higher prevalence (>2-fold) and/or abundance (inv.Ct differences of at least 2-fold) in the soil compared to leaves (P<0.05; **Figure S8A**). Only genes associated with *Staphylococcus* (n=1/1) were more prevalent (>2-fold) in the leaf samples compared to the soil samples (**Figure S8A**). Further, eleven AMR genes (aminoglycoside: aacC2 and *aadA1*; beta-lactamase group B: IMP-2, IMP-5 and IMP12; group C: ACT-1, MIR, MOX; group D: OXA-60; macrolide: *ermB* and *mefA*) were detected in more than 50% of the pooled soil, while only four AMR genes (beta-lactamase group C: ACT-1 and MIR; macrolide: *mefA* and *msrA*) were detected in more than 50% of the pooled leaf samples. Interestingly, TP7 displayed higher ARG composition similarities with TP8 and TP9 compared to TP6 (**Figure S8B**); and TP8 and TP9 displayed higher ARG composition similarities together compared with TP7 (**Figure S8B**).

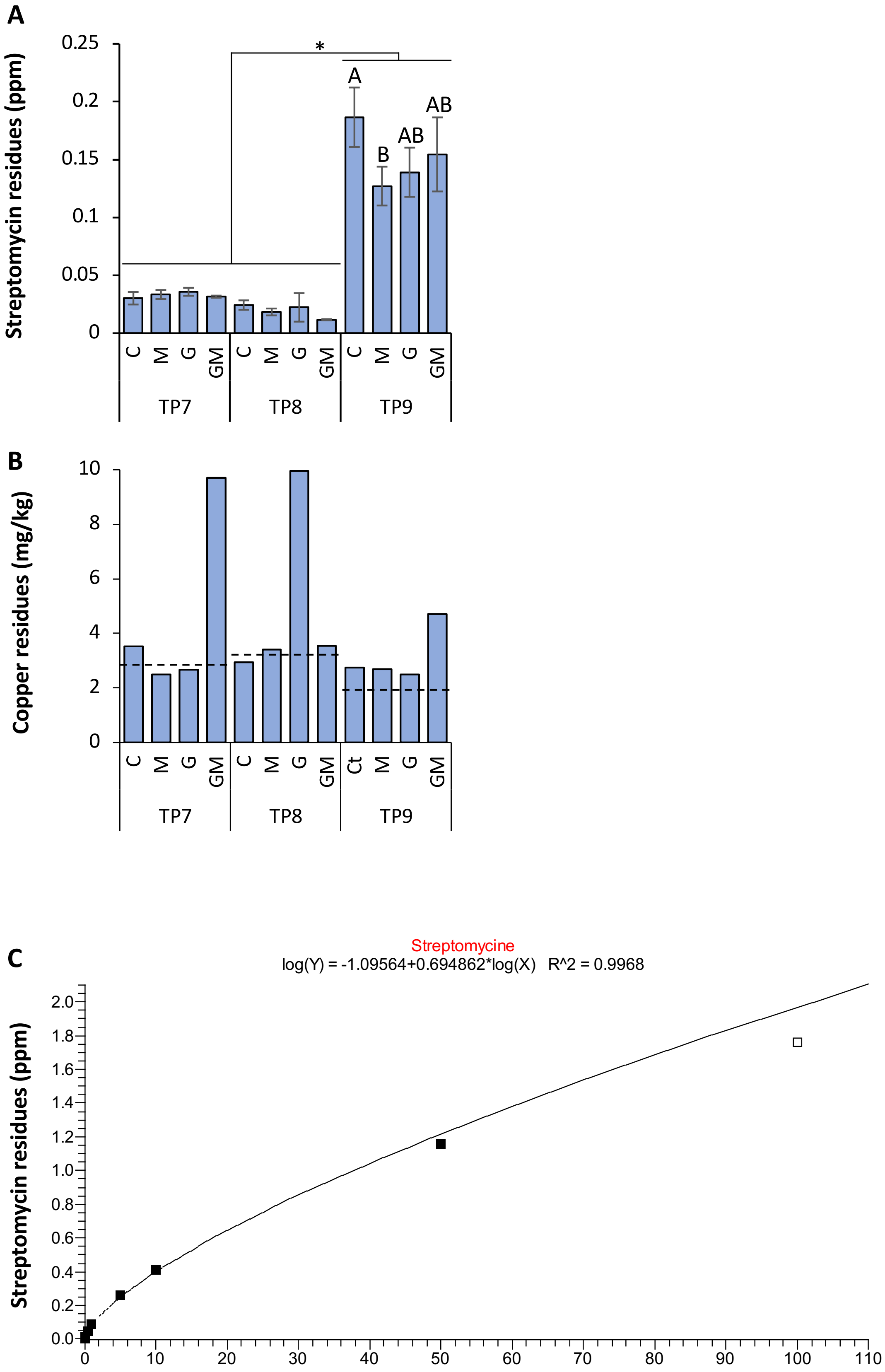
A
Streptomycin residues (ppm)
0.25
0.2
0.15
0.1
0.05
0
*
A
B
AB
AB
C
M
G
GM
TP7
TP8
TP9
B
Copper residues (mg/kg)
10
8
6
4
2
0
Ct
C
Streptomycine
log(Y) = -1.09564+0.694862*log(X) R^2 = 0.9968
Streptomycin residues (ppm)
2.0
1.8
1.6
1.4
1.2
1.0
0.8
0.6
0.4
0.2
0.0
0
10
20
30
40
50
60
70
80
90
100
110

Supplemental Figure 9. Antimicrobial residues from the soil collected between TP7 and TP9. The streptomycin (A) and copper (B) residues were measured in the soil collected from the plots treated with one of these antimicrobials during the growing season. TP7, TP8, and TP9: 6 days after the first, fourth, ad fifth antimicrobial applications, respectively. C: control (not treated); M, G, GM: rows treated once with manure and/or glyphosate. Bar: standard deviation. Dotted lines represent the average of copper residues detected in the soil of the plot not treated with copper (2.78 ±1.02 on TP7; 3.30 ±1.14 on TP8; 1.92 ±0.57 on TP9). N= 4 soil samples per group for streptomycin residues and n=1 pooled soil sample per group for copper residues. C) Standard curve of the Streptomycin residues in soil using liquid chromatography mass spectrometry (LC-MS/MS).

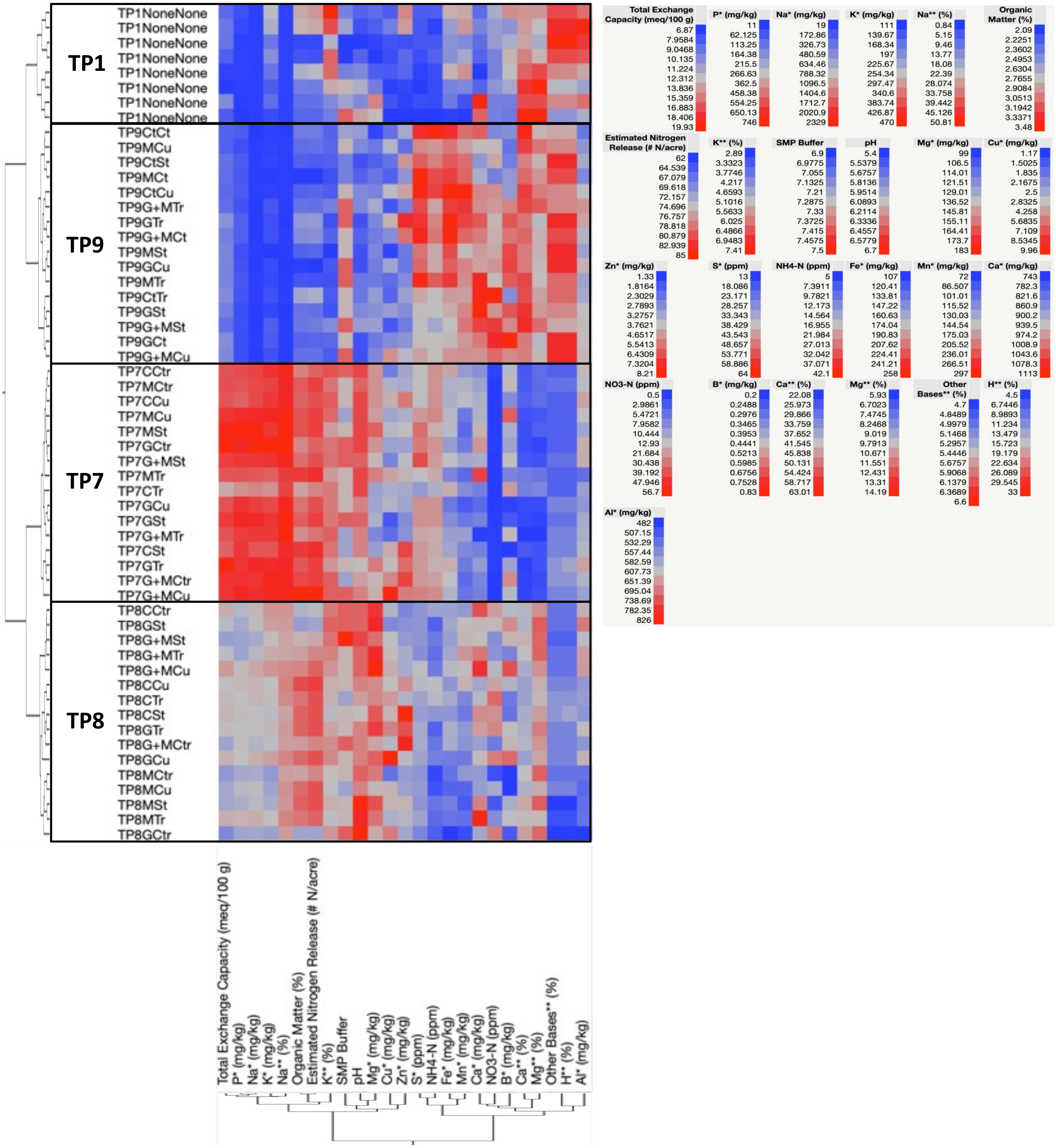

TP1
TP9
TP7
TP8
TP1NoneNone
TP1NoneNone
TP1NoneNone
TP1NoneNone
TP1NoneNone
TP1NoneNone
TP1NoneNone
TP1NoneNone
TP9CtCt
TP9MCu
TP9CtSt
TP9MCt
TP9CtCu
TP9G+MTr
TP9GTr
TP9G+MCt
TP9MSt
TP9GCu
TP9MTr
TP9CtTr
TP9GSt
TP9G+MSt
TP9GCt
TP9G+MCu
TP7CCtr
TP7MCtr
TP7CCu
TP7MCu
TP7MSt
TP7GCtr
TP7G+MSt
TP7MTr
TP7CTr
TP7GCu
TP7GSt
TP7G+MTr
TP7CSt
TP7GTr
TP7G+MCtr
TP7G+MCu
TP8CCtr
TP8GSt
TP8G+MSt
TP8G+MTr
TP8G+MCu
TP8CCu
TP8CTr
TP8CSt
TP8GTr
TP8G+MCtr
TP8GCu
TP8MCtr
TP8MCu
TP8MSt
TP8MTr
TP8GCtr
Total Exchange Capacity (meq/100 g)
P* (mg/kg)
Na* (mg/kg)
K* (mg/kg)
Na** (%)
Organic Matter (%)
Estimated Nitrogen Release (# N/acre)
K** (%)
SMP Buffer
pH
Mg* (mg/kg)
Cu* (mg/kg)
Zn* (mg/kg)
S* (ppm)
NH4-N (ppm)
Fe* (mg/kg)
Mn* (mg/kg)
Ca* (mg/kg)
NO3-N (ppm)
B* (mg/kg)
Ca** (%)
Mg** (%)
Other Bases** (%)
H** (%)
Al* (mg/kg)
Total Exchange Capacity (meq/100 g): 6.87 7.9584 9.0468 10.135 11.224 12.312 13.836 15.359 16.883 18.406 19.93
P* (mg/kg): 11 62.125 113.25 164.38 215.5 266.63 362.5 458.38 554.25 650.13 746
Na* (mg/kg): 19 172.86 326.73 480.59 634.46 788.32 1096.5 1404.6 1712.7 2020.9 2329
K* (mg/kg): 111 139.67 168.34 197 225.67 254.34 297.47 340.6 383.74 426.87 470
Na** (%): 0.84 5.15 9.46 13.77 18.08 22.39 28.074 33.758 39.442 45.126 50.81
Organic Matter (%): 2.09 2.2251 2.3602 2.4953 2.6304 2.7655 2.9084 3.0513 3.1942 3.3371 3.48
Estimated Nitrogen Release (# N/acre): 62 64.539 67.079 69.618 72.157 74.696 76.757 78.818 80.879 82.939 85
K** (%): 2.89 3.3323 3.7746 4.217 4.6593 5.1016 5.5633 6.025 6.4866 6.9483 7.41
SMP Buffer: 6.9 6.9775 7.055 7.1325 7.21 7.2875 7.33 7.3725 7.415 7.4575 7.5
pH: 5.4 5.5379 5.6757 5.8136 5.9514 6.0893 6.2114 6.3336 6.4557 6.5779 6.7
Mg* (mg/kg): 99 106.5 114.01 121.51 129.01 136.52 145.81 155.11 164.41 173.7 183
Cu* (mg/kg): 1.17 1.5025 1.835 2.1675 2.5 2.8325 4.258 5.6835 7.109 8.5345 9.96
Zn* (mg/kg): 1.33 1.8164 2.3029 2.7893 3.2757 3.7621 4.6517 5.5413 6.4309 7.3204 8.21
S* (ppm): 13 18.086 23.171 28.257 33.343 38.429 43.543 48.657 53.771 58.886 64
NH4-N (ppm): 5 7.3911 9.7821 12.173 14.564 16.955 21.984 27.013 32.042 37.071 42.1
Fe* (mg/kg): 107 120.41 133.81 147.22 160.63 174.04 190.83 207.62 224.41 241.21 258
Mn* (mg/kg): 72 86.507 101.01 115.52 130.03 144.54 175.03 205.52 236.01 266.51 297
Ca* (mg/kg): 743 782.3 821.6 860.9 900.2 939.5 974.2 1008.9 1043.6 1078.3 1113
NO3-N (ppm): 0.5 2.9861 5.4721 7.9582 10.444 12.93 21.684 30.438 39.192 47.946 56.7
B* (mg/kg): 0.2 0.2488 0.2976 0.3465 0.3953 0.4441 0.5213 0.5985 0.6756 0.7528 0.83
Ca** (%): 22.08 25.973 29.866 33.759 37.652 41.545 45.838 50.131 54.424 58.717 63.01
Mg** (%): 5.93 6.7023 7.4745 8.2468 9.019 9.7913 10.671 11.551 12.431 13.31 14.19
Other Bases** (%): 4.7 4.8489 4.9979 5.1468 5.2957 5.4446 5.6757 5.9068 6.1379 6.3689 6.6
H** (%): 4.5 6.7446 8.9893 11.234 13.479 15.723 19.179 22.634 26.089 29.545 33
Al* (mg/kg): 482 507.15 532.29 557.44 582.59 607.73 651.39 695.04 738.69 782.35 826

Supplemental Figure 10. Physico-chemical properties of the soil collected on TP1, TP7, TP8, and TP9. The two-way clusterization plot was generated based on the physico-chemical properties recorded from the soil collected from plots treated with specific health and disease management practice combinations during four strategic time points. TP1: 1 day before the beginning of the experiment, TP7, TP8, and TP9: 6 days after the first, fourth, and fifth antimicrobial applications, respectively. Ct: control (not treated); M, G, G+M: rows treated once with manure and/or glyphosate. Cu, St, and Tr: experimental plots treated weekly with copper, streptomycin, or triazole.
