# Supplementary material for: Dairy manure, glyphosate, and antimicrobials (copper, streptomycin, and triazole) modulated the composition of antimicrobial resistance at the gene and microbial levels in a processing tomato field
Source: Microbiol Spectr. 2026 Mar 17;14(4):e02003-25. doi: 10.1128/spectrum.02003-25 (PMC13055215; doi:10.1128/spectrum.02003-25)
Supplement: Figure S3 — Impact of glyphosate and manure on the levels of culturable antibiotic resistant bacteria in the soil and leaf samples collected between TP4 and TP9. [file spectrum.02003-25-s0002.pdf]

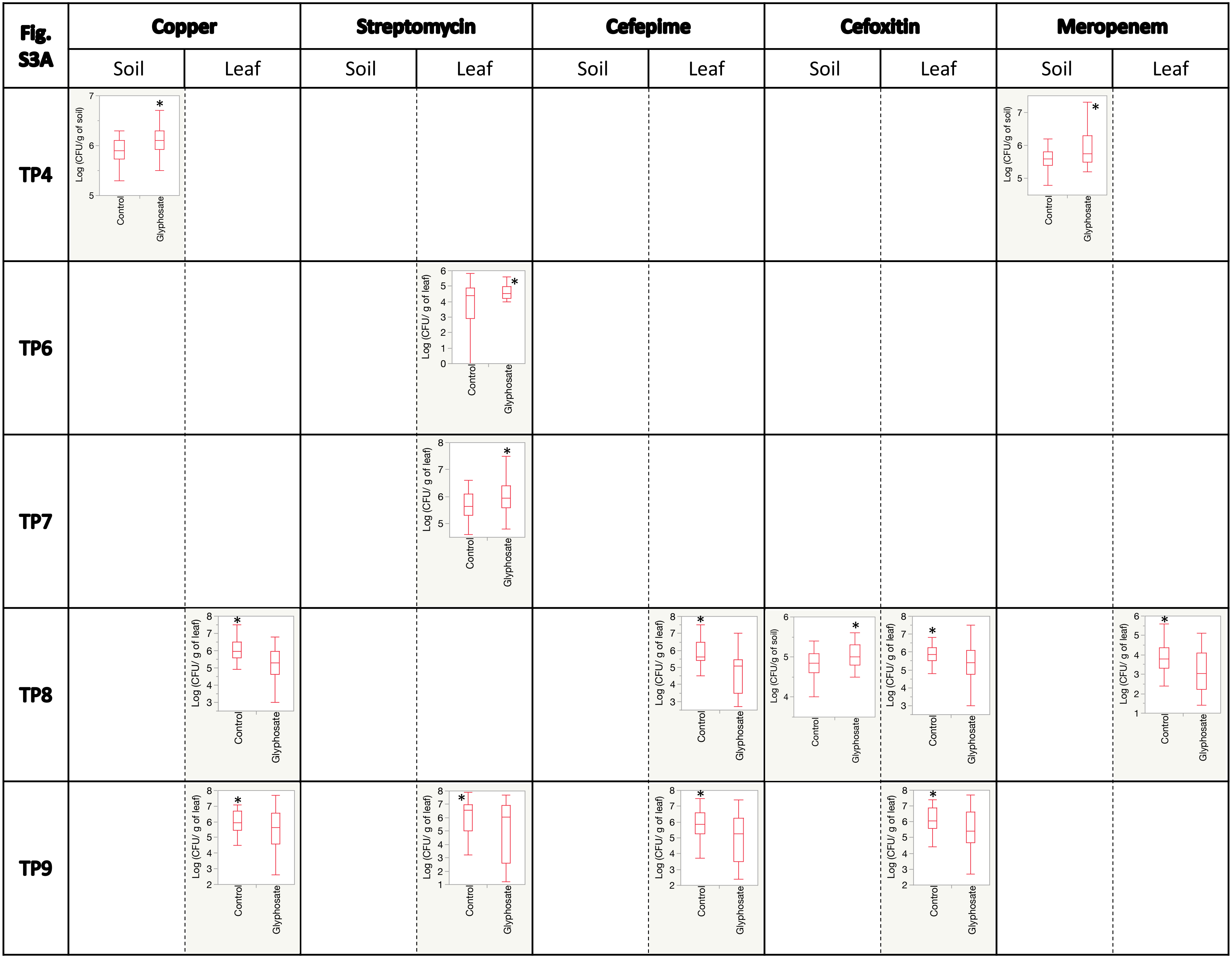
Fig. S3A
Copper
Streptomycin
Cefepime
Cefoxitin
Meropenem
Soil
Leaf
TP4
TP6
TP7
TP8
TP9
Log (CFU/g of soil)
Log (CFU/ g of leaf)
Control
Glyphosate
*

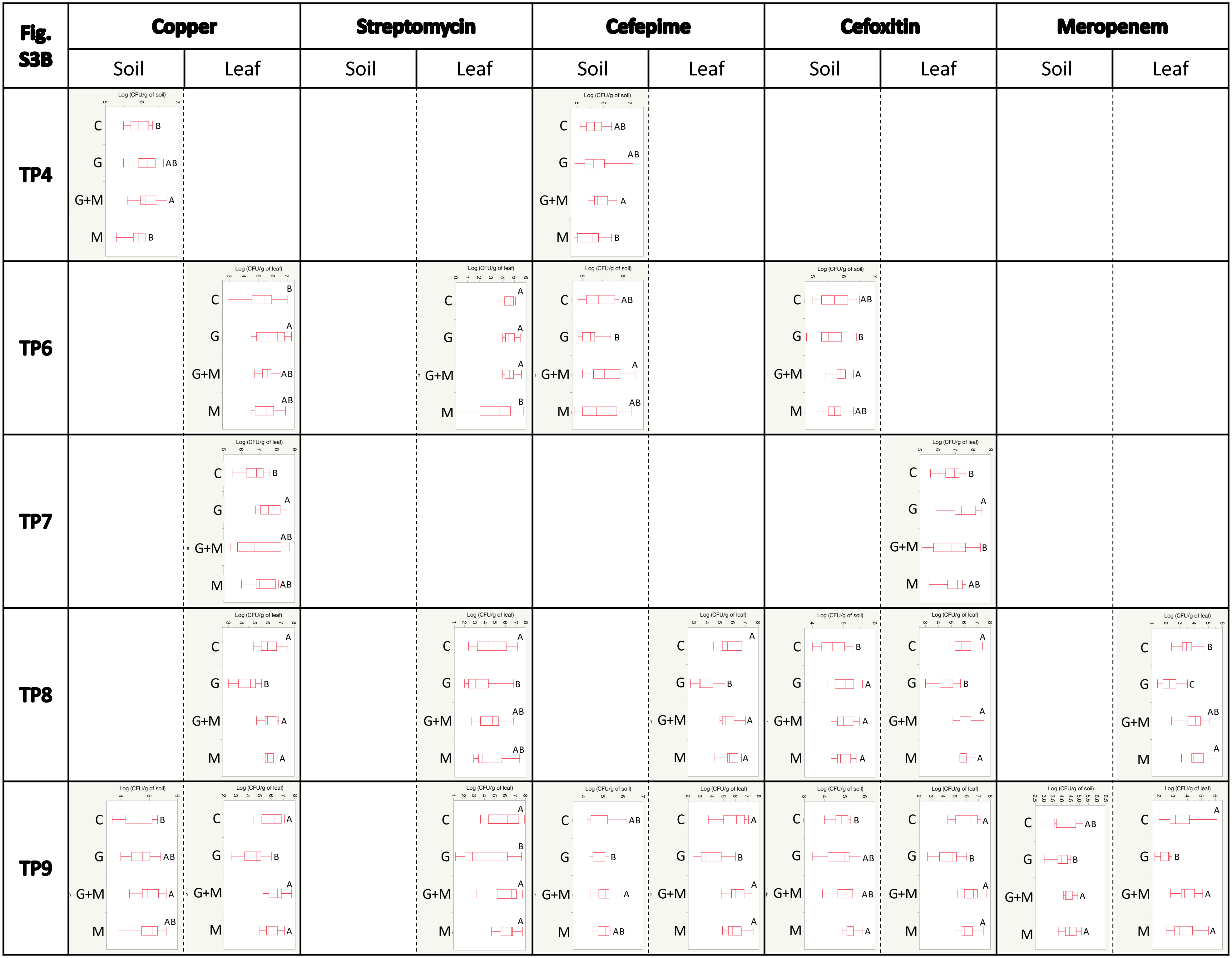

Fig. S3B
Copper
Streptomycin
Cefepime
Cefoxitin
Meropenem
Soil
Leaf
TP4
TP6
TP7
TP8
TP9
Log (CFU/g of soil)
Log (CFU/g of leaf)
C
G
G+M
M

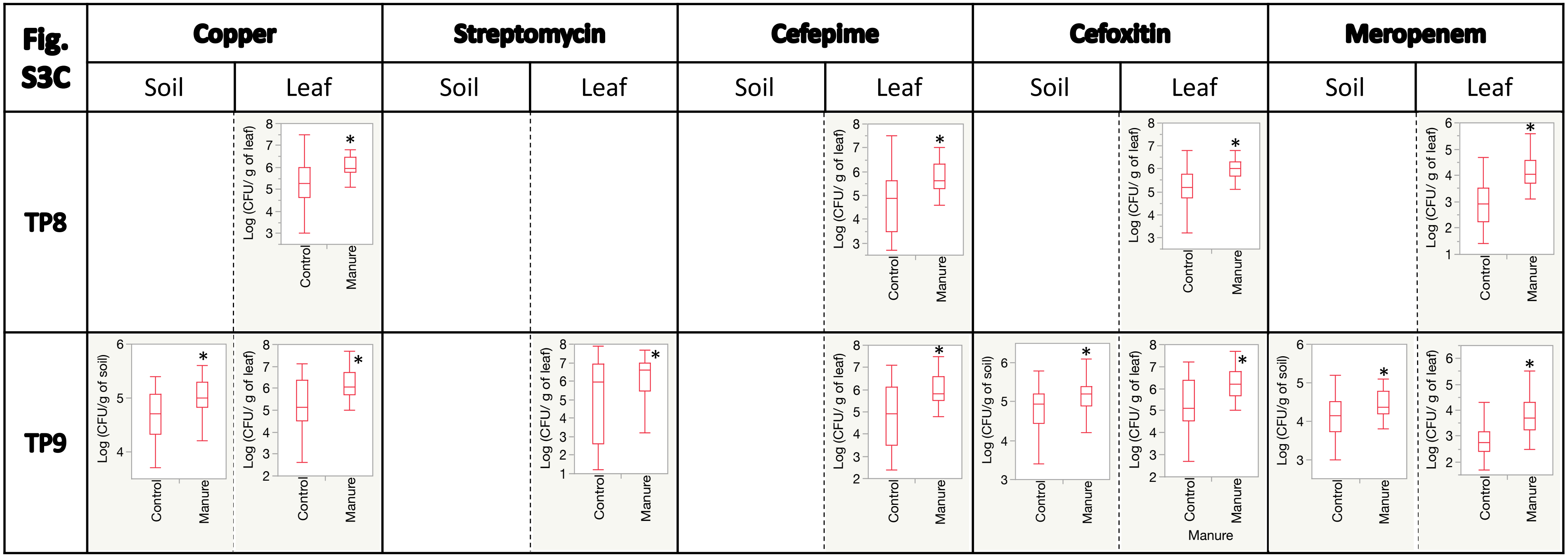

Supplemental Figure 3. Impact of glyphosate and manure on the levels of culturable antibiotic resistant bacteria in the soil and leaf samples collected between TP4 and TP9. Quantification of culturable bacteria resistant to 200 µg/ml copper, 200 µg/ml streptomycin, 4 µg/ml cefepime, 8 µg/ml cefoxitin, and 1 µg/ml of meropenem by direct plating on Mac Conkey agar. Letters represent statistical categories (P<0.05). C (control): non-treated rows; G (glyphosate), M (manure), and GM: rows applied with manure and/or glyphosate. N= 32 pooled samples per group in fig. S2A and S2C, and 16 pooled samples per group in fig. S2B. Star: the bacterial level was significantly higher in the designated group compared to the other group (P<0.05; n= 8 seedlings).
