# Supplementary material for: Dairy manure, glyphosate, and antimicrobials (copper, streptomycin, and triazole) modulated the composition of antimicrobial resistance at the gene and microbial levels in a processing tomato field
Source: Microbiol Spectr. 2026 Mar 17;14(4):e02003-25. doi: 10.1128/spectrum.02003-25 (PMC13055215; doi:10.1128/spectrum.02003-25)
Supplement: Table S1 — Agricultural practices and samples' collection. [file spectrum.02003-25-s0003.docx]

Supplemental Table 1. Agricultural practices and sample collection

| **Management practices** | **Input 1** | **Input 2** | **Input 3** | **Timeline (days)** | **Sample collection time point (TP)** | Soil | Leaf | Manure | Seedling |
| --- | --- | --- | --- | --- | --- | --- | --- | --- | --- |
|  | | | | 1 | TP1 | 320 |  |  |  |
| Manure application | Squeezed dairy manure |  |  | 2 | TP2 |  |  | 8 |  |
|  | | | | 86 | TP3 | 320 |  |  |  |
| Glyphosate | Roundup Weathermax 3.5 L/ha |  |  | 87 |  |  |  |  |  |
|  | | | | 91 | TP4 |  |  |  |  |
| Transplantation | 6-week-old Peto seedlings |  |  | 92 | TP5 |  |  |  | 8 |
|  | | | | 96 | TP6 | 320 | 320 |  |  |
| First antimicrobial application | Kocide 2000 1.68 kg/ha | Harbour 200 µg/l | Propimax EC 0.35 l/ha | 97 |  |  |  |  |  |
|  | | | | 105 | TP7 | 320 | 320 |  |  |
| Second antimicrobial application | Kocide 2000 1.68 kg/ha | Harbour 200 µg/l | Propimax EC 0.35 l/ha | 106 |  |  |  |  |  |
| Third antimicrobial application | Kocide 2000 1.68 kg/ha | Harbour 200 µg/l | Propimax EC 0.35 l/ha | 113 |  |  |  |  |  |
| Fourth antimicrobial application | Kocide 2000 1.68 kg/ha | Harbour 200 µg/l | Propimax EC 0.35 l/ha | 119 |  |  |  |  |  |
|  | | | | 125 | TP8 | 320 | 320 |  |  |
| Fifth antimicrobial application | Kocide 2000 1.68 kg/ha | Harbour 200 µg/l | Propimax EC 0.35 l/ha | 126 |  |  |  |  |  |
|  | | | | 131 | TP9 | 320 | 320 |  |  |
| **Total number of samples collected** | | | | | | **1920** | **1280** | **8** | **8** |
