# Supplementary material for: Dairy manure, glyphosate, and antimicrobials (copper, streptomycin, and triazole) modulated the composition of antimicrobial resistance at the gene and microbial levels in a processing tomato field
Source: Microbiol Spectr. 2026 Mar 17;14(4):e02003-25. doi: 10.1128/spectrum.02003-25 (PMC13055215; doi:10.1128/spectrum.02003-25)
Supplement: Table S2 — Details about the Microbial DNA qPCR Array used for the detection of antibiotic resistance genes. [file spectrum.02003-25-s0004.docx]

Supplemental Table 2. Details of the Microbial DNA qPCR Array used for the detection of antibiotic resistance genes.

| **Antibiotic classification / virulence factor gene description** | **Species/gene** | **Also detects / associated species (virulence factor gene)** |
| --- | --- | --- |
| Fluoroquinolone resistance | AAC(6)-Ib-cr |  |
| Aminoglycoside-resistance | aacC1 |  |
| Aminoglycoside-resistance | aacC2 |  |
| Aminoglycoside-resistance | aacC4 |  |
| Aminoglycoside-resistance | aadA1 |  |
| Aminoglycoside-resistance | aphA6 |  |
| Class A beta-lactamase | BES-1 |  |
| Class A beta-lactamase | BIC-1 |  |
| Class A beta-lactamase | CTX-M-1 Group | Detects CTX-M-1 type (37 variants) |
| Class A beta-lactamase | CTX-M-8 Group | Detects CTX-M-8 type (3 variants) |
| Class A beta-lactamase | CTX-M-9 Group | Detects CTX-M-9 type (40 variants) |
| Class A beta-lactamase | GES | GES,IBC |
| Class A beta-lactamase | IMI & NMC-A | NMC-A,IMI-2,IMI-3 |
| Class A beta-lactamase | KPC | KPC-1,KPC-2,KPC-3,KPC-4,KPC-5,KPC-6,KPC-7,KPC-8,KPC-9,KPC-10,KPC-11 |
| Class A beta-lactamase | Per-1 group | Per-1,Per-3,Per-4,Per-5 |
| Class A beta-lactamase | Per-2 group | Per-2,Per-6 |
| Class A beta-lactamase | SFC-1 |  |
| Class A beta-lactamase | SFO-1 |  |
| Class A beta-lactamase | SHV |  |
| Class A beta-lactamase | SHV(156D) |  |
| Class A beta-lactamase | SHV(156G) |  |
| Class A beta-lactamase | SHV(238G240E) |  |
| Class A beta-lactamase | SHV(238G240K) |  |
| Class A beta-lactamase | SHV(238S240E) |  |
| Class A beta-lactamase | SHV(238S240K) |  |
| Class A beta-lactamase | SME | SME-1,SME-2,SME-3 |
| Class A beta-lactamase | TLA-1 |  |
| Class A beta-lactamase | VEB | VEB-1,VEB-2,VEB-3,VEB-4,VEB-5,VEB-6,VEB-7 |
| Class B beta-lactamase | ccrA |  |
| Class B beta-lactamase | IMP-1 group | IMP-1,IMP-3,IMP-4,IMP-6,IMP-10,IMP-25,IMP-26 |
| Class B beta-lactamase | IMP-12 group | IMP-12,IMP-14,IMP-16,IMP-18 |
| Class B beta-lactamase | IMP-2 group | IMP-2,IMP-8,IMP-11,IMP-19,IMP-20,IMP-21,IMP-24 |
| Class B beta-lactamase | IMP-5 group | IMP-5,IMP-7,IMP-9,IMP-13,IMP-15,IMP-22 |
| Class B beta-lactamase | NDM | NDM-1,NDM-2 |
| Class B beta-lactamase | VIM-1 group | VIM-1,VIM-2,VIM-3,VIM-4,VIM-5,VIM-6,VIM-8,VIM-9,VIM-10,VIM-11,VIM-12,VIM-14,VIM-15,VIM-16,VIM-17,VIM-18,VIM-19,VIM-20,VIM-23,VIM-24,VIM-25,VIM-26 |
| Class B beta-lactamase | VIM-13 | VIM-13 (28 variants) |
| Class B beta-lactamase | VIM-7 |  |
| Class C beta-lactamase | ACC-1 group | ACC-1,ACC-2,ACC-4 |
| Class C beta-lactamase | ACC-3 |  |
| Class C beta-lactamase | ACT 5/7 group | ACT-5,ACT-7 |
| Class C beta-lactamase | ACT-1 group | ACT-1,ACT-2,ACT-3,ACT-4,ACT-6 |
| Class C beta-lactamase | CFE-1 |  |
| Class C beta-lactamase | CMY-10 Group | CMY-1,CMY-8,CMY-9,CMY-10,CMY-19 |
| Class C beta-lactamase | DHA | DHA-1,DHA-2,DHA-3,DHA-5,DHA-6,DHA-7 |
| Class C beta-lactamase | FOX | FOX-1,FOX-2,FOX-3,FOX-4,FOX-5,FOX-6,FOX-7 |
| Class C beta-lactamase | LAT | LAT-1,LAT-3,LAT-4,CMY-2 group |
| Class C beta-lactamase | MIR | MIR-1,MIR-2,MIR-3,MIR-4,MIR-5 |
| Class C beta-lactamase | MOX | MOX-1,MOX-2,MOX-3,MOX-4,MOX-5,MOX-6,MOX-7 |
| Class D beta-lactamase | OXA-10 Group | OXA-10,OXA-11,OXA-14,OXA-16,OXA-17,OXA-19,OXA-28,OXA-35,OXA-142,OXA-145,OXA-147 |
| Class D beta-lactamase | OXA-18 |  |
| Class D beta-lactamase | OXA-2 Group | OXA-2,OXA-15,OXA-32,OXA-34,OXA-141,OXA-161 |
| Class D beta-lactamase | OXA-23 Group | OXA-23,OXA-27,OXA-49,OXA-73,OXA-133,OXA-146,OXA-165,OXA-166,OXA-167,OXA-168,OXA-169,OXA-170,OXA-171 |
| Class D beta-lactamase | OXA-24 Group | OXA-24,OXA-25,OXA-26,OXA-40,OXA-72,OXA-139,OXA-160 |
| Class D beta-lactamase | OXA-45 |  |
| Class D beta-lactamase | OXA-48 Group | OXA-48,OXA-162,OXA-163,OXA-181 |
| Class D beta-lactamase | OXA-50 Group | OXA-50 group (50 variants) |
| Class D beta-lactamase | OXA-51 Group | OXA-51 group (65 variants) |
| Class D beta-lactamase | OXA-54 |  |
| Class D beta-lactamase | OXA-55 | OXA-55,OXA-SH |
| Class D beta-lactamase | OXA-58 Group | OXA-58,OXA-96,OXA-97,OXA-164 |
| Class D beta-lactamase | OXA-60 | OXA-60,OXA-60a,OXA-60b,OXA-60c |
| Erythromycin resistance | ereB |  |
| Fluoroquinolone resistance | QepA | QepA1,QepA2 |
| Fluoroquinolone resistance | QnrA | QnrA1,QnrA2,QnrA3,QnrA4,QnrA5,QnrA6,QnrA7 |
| Fluoroquinolone resistance | QnrB-1 group | QnrB1,QnrB2,QnrB3,QnrB6,QnrB7,QnrB9,QnrB13,QnrB14,QnrB15,QnrB16,QnrB17,QnrB18,QnrB20,QnrB23,QnrB24,QnrB29,QnrB30 |
| Fluoroquinolone resistance | QnrB-31 group | QnrB31,QnrB32 |
| Fluoroquinolone resistance | QnrB-4 group | QnrB4,QnrB11,QnrB12,QnrB22 |
| Fluoroquinolone resistance | QnrB-5 group | QnrB5,QnrB10,QnrB19 |
| Fluoroquinolone resistance | QnrB-8 group | QnrB8,QnrB21,QnrB25,QnrB27,QnrB28 |
| Fluoroquinolone resistance | QnrC |  |
| Fluoroquinolone resistance | QnrD |  |
| Fluoroquinolone resistance | QnrS | QnrS1,QnrS2,QnrS3,QnrS4 |
| Macrolide Lincosamide Streptogramin_b | ermA |  |
| Macrolide Lincosamide Streptogramin_b | ermB |  |
| Macrolide Lincosamide Streptogramin_b | ermC |  |
| Macrolide Lincosamide Streptogramin_b | mefA |  |
| Macrolide Lincosamide Streptogramin_b | msrA |  |
| Multidrug resistance efflux pump | oprj |  |
| Multidrug resistance efflux pump | oprm |  |
| Tetracycline efflux pump | tetA |  |
| Tetracycline efflux pump | tetB |  |
| Vancomycin resistance | vanB |  |
| Vancomycin resistance | vanC |  |
| Staphylococcus aureus | Staphylococcus aureus |  |
| Beta-lactam resistance | mecA |  |
| Panton-Valentine leukocidin chain F precursor | lukF | Staphylococcus aureus |
| Immunoglobulin G binding protein A precursor | spa | Staphylococcus aureus |

Source: This document was downloaded from the QIAGEN website (https://www.qiagen.com/us/products/discovery-and-translational-research/pcr-qpcr/qpcr-assays-and-instruments/microbial-dna-qpcr-assays-and-panels/microbial-dna-qpcr-arrays/#productdetails)
