# Supplementary material for: Dairy manure, glyphosate, and antimicrobials (copper, streptomycin, and triazole) modulated the composition of antimicrobial resistance at the gene and microbial levels in a processing tomato field
Source: Microbiol Spectr. 2026 Mar 17;14(4):e02003-25. doi: 10.1128/spectrum.02003-25 (PMC13055215; doi:10.1128/spectrum.02003-25)
Supplement: Table S3 — Antimicrobial resistant gene profile from the soil collected on TP1 and the manure applied on the field at TP2. [file spectrum.02003-25-s0005.docx]

**Supplemental Table 3. Antimicrobial resistant gene profile from the soil collected on TP1 and the manure applied on the field at TP2.**

**A. Soil collected at TP1**

| **Antibiotic classification** | **Gene** | **Inv.Ct** |
| --- | --- | --- |
| Aminoglycoside-resistance | *aacC1* | 3 |
|  | *aacC2* | 2 |
|  | *aadA1* | 5 |
| Macrolide Lincosamide Streptogramin b | *ermA* | 1 |
|  | *ermB* | 0 |
|  | *mefA* | 4 |
| Class B beta-lactamase | IMP-12 group | 1 |
|  | IMP-5 group | 4 |
| Class D beta-lactamase | IMP-2 group | 1 |
| Tetracycline efflux pump | *tetA* | 3 |

**B. Dairy manure sample collected before application on the field (TP2)**

| **Antibiotic classification** | **Gene** | **Inv.Ct** |
| --- | --- | --- |
| Aminoglycoside-resistance | *aadA1* | 6 |
| Erythromycin resistance | *ereB* | 5 |
| Macrolide Lincosamide Streptogramin b | *ermA* | 3 |
|  | *ermB* | 7 |
|  | *mefA* | 11 |
| Class A beta-lactamase | VEB | 3 |
| Class D beta-lactamase | OXA-2 group | 2 |
|  | OXA-58 group | 5 |
| Tetracycline efflux pump | *tetA* | 7 |
|  | *tetB* | 7 |

Inv.Ct (inverted Ct value) is an estimation of the selected gene abundance in the designated sample. Inv.Ct is calculated using the following formula (total number of cycles [40] - Ct value). Each real-time PCR were performed using the same amount of DNA (10.5 ng per reaction, as recommended by the manufacturer).
