# Supplementary material for: Dairy manure, glyphosate, and antimicrobials (copper, streptomycin, and triazole) modulated the composition of antimicrobial resistance at the gene and microbial levels in a processing tomato field
Source: Microbiol Spectr. 2026 Mar 17;14(4):e02003-25. doi: 10.1128/spectrum.02003-25 (PMC13055215; doi:10.1128/spectrum.02003-25)
Supplement: Table S4 — Microbiome composition differences detected between the agricultural practices at the genus/species levels. [file spectrum.02003-25-s0006.docx]

Supplemental Table 4 . Microbiome composition differences detected between the agricultural practices at the genus/species levels

Alpha diversity analysis based on Faith’s Phylogenetic Diversity (PD) and Shannon index revealed temporal and treatment-dependent differences in microbial community structure across soil and leaf samples. In soil samples, diversity fluctuated over time following the application of glyphosate and dairy manure (TP3–TP9). At TP4, both indices showed significant variation (P < 0.01), with the highest diversity observed in glyphosate-only plots (G) and the lowest in control plots (C). Similar patterns persisted at TP6 and TP7, where manure (M) and combined glyphosate-manure (GM) treatments maintained higher microbial diversity compared to controls, whereas no significant differences were detected at TP8. By TP9, soil communities again showed distinct clustering, with decreasing diversity from G to M plots. In contrast, leaf-associated microbiomes exhibited generally lower diversity values, ranging from 3–31 for Faith’s PD and 1.0–3.7 for Shannon index, but displayed clearer treatment effects. Leaf samples collected after glyphosate and manure applications (TP6–TP9) consistently showed higher diversity in manure-treated plots compared to controls. For antimicrobial-treated plots (copper, streptomycin, and propiconazole), soil alpha diversity decreased significantly at TP7 and TP8, with controls exhibiting greater richness than copper- and streptomycin-treated soils. By TP9, no significant differences were observed, suggesting a temporal recovery of the soil microbiome. Leaf microbiomes, however, remained more sensitive to treatments: at TP7, both Faith’s PD and Shannon indexes were highest in control plants and lowest in streptomycin-treated leaves, with intermediate values in triazole- and copper-treated samples. Differences in leaf microbial diversity diminished over time, and by TP9, only minor distinctions persisted across treatments. Collectively, these results indicate that both herbicide-manure combinations and antimicrobial treatments temporarily altered microbial diversity, with stronger effects observed on phyllosphere communities than in soil environments.

| At TP7, three distinct microbiome profiles were identified between the plots applied with manure or glyphosate (C, G, GM, and M) in soil samples. More precisely, plots applied with manure only (M group) harbored the highest relative abundance in Acidobacteria (uncultured bacterium 213 [only detected in M group; 0.4%]), Bacteroidetes (*Cytophaga* [2.1-fold], *OLB12* [1.8-fold] and *Ohtaekwangia* [1.5-fold]), Chloroflexi (*Herpetosiphon* [6.5-fold]), Proteobacteria (*Skermanella* [2-fold], *Caulobacter* [2-fold], *Tardiphaga* [18.5-fold], *Rhodobacteraceae* [1.4-fold], *Altererythrobacter* [1.8-fold], *Peredibacter* [5-fold], *Blrii41* [1.8-fold] and *Cellvibrio* [2-fold]), and Verrucomicrobia (*Lacunisphaera* [1.9-fold]) in the soil compared to the other groups (C, G and GM groups; P<0.01), while Acidobacteria (*Bryobacter* [1.5-fold]) and Chloroflexi (HSB OF53-F07 [2-fold]) displayed the opposite trends. plots applied with manure and glyphosate (GM group) harbored the lowest relative abundance in Bacteroidetes (*Emticicia* [3.5-fold]), Proteobacteria (*Methylotenera* [2.7-fold]), and Verrucomicrobia (*Methylacidiphilaceae* [8-fold]) in the soil compared to the other groups (C, G and M groups; P<0.01), while Firmicutes (*Tumebacillus* [3-fold]), Plantomycetes (WD2101 soil group [1.3-fold]) and Nitrospirae (*Nitrospira japonica* [2.1-fold]) displayed the opposite trend. Non-treated plots (C group) harbored the highest relative abundance in Actinobacteria (CL500-29 marine group [6-fold] and *Arthrobacter* [only detected in C group; 0.1%]), Firmicutes (*Bacillus* [2-fold]), Plantomycetes (*Pirellula* sp. Br1-4 [only detected in C group; 0.04%]), Proteobacteria (*Massilia* [1.4-fold] and *Dokdonella* *ginsengisoli* [4.7-fold]), and Verrucomicrobia (*Candidatus Udaeobacter* [1.6-fold]) in the soil compared to the other groups (G, GM and M groups; P<0.01). The abundance of Acidobacteria bacterium 13_2_20CM_56_17 was significantly higher (6-fold) in glyphosate applied plots (G and GM groups) compared to the other groups (C and M groups; P<0.01). In addition, two distinct microbiome profiles in soil samples at TP7 were identified between the pesticide used (Ct, Cu, St, and Tr). More precisely, non-treated plots (Ct group) harbored the highest relative abundance in Acidobacteria (uncultured bacterium DA023 [5.5-fold]) and Proteobacteria (P3OB-42 [10-fold]) compared to the other groups (Cu, St and Tr groups; P<0.01). Plots treated with triazole (Tr group) harbored the highest relative abundance in Acidobacteria (*Catenulisporales* [16.7-fold]) and Proteobacteria (*Micropepsaceae* [1.3-fold]) compared to the other groups (Ct, Cu and St groups; P<0.01). At TP7 in the leaf tissues, non-treated plots (C group) harbored the highest relative abundance in Bacteroidetes (*Microscillaceae* [11.5-fold]) and Proteobacteria (BIrii41 [13.7-fold], *Duganella* [37.5-fold] and *Pseudomonas* [18.7-fold]) compared to the other groups (G, GM and M groups; P<0.01). Similarly, at TP7 in the leaf tissues, non-treated plots (C group) harbored the highest relative abundance in Actinobacteria (*Propionibacteriales* [8.4-fold], *Mycobacterium* [only detected in C group; 0.04%], *Geodermatophilaceae* [3.8-fold], *Intrasporangiaceae* [3.5-fold] and *Gaiellales* [21.3-fold]) and Proteobacteria (*Rickettsiales* [2.2-fold]) compared to the other groups (Cu, St and Tr groups; P<0.01). |
| --- |
| At TP8, five distinct microbiome profiles were identified between the plots applied with manure or glyphosate (C, G, GM, and M) in soil samples. More precisely, plots applied with manure only (M group) harbored the highest relative abundance in Acidobacteria (uncultured eubacterium WD283 [2.1-fold] and uncultured bacterium 259 [only detected in M group; 0.08%]), Actinobacteria (*Ilumatobacter* [2.6-fold] and *Blastococcus* [5.2-fold]), Bacteroidetes (*Terrimonas* [1.7-fold]) and Proteobacteria (*Skermanella* [2.6-fold] and *Dongia* *mobilis* [26.7-fold]) compared to the other groups (C, G, and GM groups; P<0.01), while Acidobacteria (*Bryobacter* [1.3-fold]) and Proteobacteria (*Micropepsaceae* [1.6-fold] and *Luteimonas* [3.2-fold]) displayed the opposite trend. plots applied with manure (M and GM groups) harbored the highest relative abundance in Acidobacteria (*Paludibaculum* [1.8-fold]), Bacteroidetes (NS9 marine group [3.4-fold]), Planctomyetes (uncultured *Verrucomicrobium* DEV009 [3.6-fold]), Proteobacteria (*Brevundimonas* [only detected in M and GM groups, 0.03% and 0.05%, respectively], *Altererythrobacter* [1.9-fold] and R7C24 [2.3-fold]) and Verrucomicrobia (*Lacunisphaera* [1.7-fold]) compared to the other groups (C and G groups; P<0.01), while Proteobacteria (MND1 [2.1-fold] and *Arenimonas daechungensis* [only detected in C and G groups, 0.2%]) and Verrucomicrobia (ADurb.Bin063-1 [1.5-fold]) displayed the opposite trend. Non-treated plots (C group) harbored the highest relative abundance in Acidobacteria (*Corynebacteriales* [2.1-fold]), Bacteroidetes (JG30-B11 [3.6-fold]), Gemmatimonadetes (uncultured bacterium KF-JG30-B11 [1.4-fold]), Patescibacteria (*Candidatus* *Magasanikbacteria* [only detected in C group; 0.02%]) and Proteobacteria (*Micavibrionaceae* [18.4-fold], *Syntrophaceae* [7.5-fold], WD260 [9.3-fold], *Massilia* [1.7-fold], *Chujaibacter* [4.7-fold] and *Rhodanobacter* [1.5-fold]) compared to the other groups (G, GM and M groups; P<0.01), while Bacteroidetes (*Ohtaekwangia* [2.2-fold]) and Verrucomicrobia (*Verrucomicrobiaceae* [2.7-fold]) displayed the opposite trend; plots applied with glyphosate only (G group) harbored the highest relative abundance in Actinobacteria (*Thermoleophilia* [1.5-fold] and *Kribbella* [6.2-fold]), Planctomyetes (CPla-3 termite group [2-fold]) and Proteobacteria (RCP2-54 [3-fold]) compared to the other groups (C, GM and M groups; P<0.01); plots applied with manure and glyphosate (GM group) harbored the highest relative abundance in Bacteroidetes (*Ferruginibacter* [1.4-fold] and *Cytophagaceae* [3.3-fold]), Chloroflexi (JG30a-KF-32 [1.6-fold]), Planctomyetes (*Planctomycetales* bacterium Ellin7224 [6.2-fold]), Proteobacteria (*Methylotenera* [3.2-fold]) and Verrucomicrobia (bacterium Ellin507 [4.1-fold] and *Luteolibacter* [1.5-fold]) compared to the other groups (C, G and M groups; P<0.01), while Acidobacteria (bacterium Ellin7504 [3.1-fold]) displayed the opposite trend. Four distinct microbiome profiles in soil samples at TP8 were identified between the pesticide used (Ct, Cu, St, and Tr). Non-treated plots (Ct group) harbored the highest relative abundance in Bacteroidetes (KD3-93 [1.8-fold]), Firmicutes (*Lachnospiraceae* [70.3-fold] and *Peptostreptococcaceae* [1.9-fold]) and Proteobacteria (*Polyangium* [7.5-fold]) compared to the other groups (Cu, St and Tr groups; P<0.01); Plots applied with triazole (Tr group) harbored the highest relative abundance in Actinobacteria (*Acidimicrobiia* [2-fold]) and Proteobacteria (*Aetherobacter* [2.1], *Ectothiorhodospira* sp. enrichment culture [only detected in M group; 0.01%] and *Rudaea* [3.8-fold]) compared to the other groups (Ct, Cu and St groups; P<0.01); Plots applied with streptomycin (St group) harbored the lowest relative abundance in Proteobacteria (*Lysobacter* [4.6-fold]) and Spirochaetes (7.4-fold) compared to the other groups (Ct, Cu and Tr groups; P<0.01), while Bacteroidetes (*Lacibacter* [7.8-fold] and *Niastella yeongjuensis* [3.2-fold]) displayed the opposite trends; Plots applied with copper sulfate (Cu group) harbored the highest relative abundance in Verrucomicrobia (*Pedosphaera* [15.8-fold]), while Firmicutes (*Kurthia* [3.3-fold]) displayed the opposite trends. No significant differences were observed in the leaf tissues at TP8 between agricultural practices (P>0.01). |
| At TP9, three distinct microbiome profiles were identified between the plots applied with manure or glyphosate (C, G, GM, and M) in soil samples. More precisely, plots applied with manure only (M group) harbored the highest relative abundance in Actinobacteria (*Curtobacterium*[18.4-fold]) compared to the other groups (C, G, and GM groups; P>0.01). plots applied with glyphosate and manure only (GM group) harbored the highest relative abundance in Proteobacteria (*Massilia* [7.1-fold]) compared to the other groups (C, G, and M groups; P>0.01). Non-treated plots (C group) harbored the highest relative abundance in 39 ASV compared to the other groups (M, G, and GM groups; P>0.01). Among them, six ASV possessed a high relative abundance (>1%) in the C group; Acidobacteria (*Bryobacter* [297.2-fold] and *Candidatus* Solibacter [43.8-fold]), Firmicutes (*Bacillus* [39.7-fold]), Gemmatimonadetes (*Gemmatimonas* [139.9-fold]), Proteobacteria (*Acidibacter* [50.3-fold]) and Verrucomicrobia (*Candidatus* Udaeobacter [34-fold]) were significantly higher in C group compared to the other groups (M, G, and GM groups; P>0.01). Similarly, two distinct microbiome profiles were identified between the plots applied with manure or glyphosate (C, G, GM, and M) in leaf samples at TP9. Plots applied with glyphosate only (G group) harbored the highest relative abundance in Planctomycetes (*Zavarzinella* [9.5-fold] and *Aquisphaera* [6.3-fold]) and Proteobacteria (*Anaeromyxobacter* [5.5-fold] and MND1 [6.1-fold]) compared to the other groups (C, GM, and M groups; P>0.01). *Tahibacter* (0.12%) was only detected in the plots treated with both glyphosate and manure (GM group; P<0.01). Only *Phaselicystis* was significantly higher (3.2-fold) in the leaf tissues collected from the Triazole treated plots (Tr group) at TP9 compared to the other groups (Ct, Cu and St). No significant difference was detected in the soil samples at TP9 between pesticide used (Ct, Cu, St and Tr) at the genus or species level. |

| **Microbiome Diversity & Composition – Summary Recap** | | | | | |
| --- | --- | --- | --- | --- | --- |
| **Time Point** | **Compartment** | **Treatment Type** | **Main Observation** | **Diversity Trend** | **Notes** |
| **TP4** | Soil | Glyphosate / Manure | Significant variation (P<0.01) | **Highest:** Glyphosate; **Lowest:** Control | Early fluctuation after application |
| **TP6–TP7** | Soil | Manure & GM | Higher diversity than control | Increase diversity | Strong treatment effect |
| **TP8** | Soil | Manure / Glyphosate | No significant differences | ≈ Stable | Temporary normalization |
| **TP9** | Soil | All | Distinct clustering again |  | Partial recovery pattern |
| **TP6–TP9** | Leaf | Manure | Clear treatment effect | Increased diversity vs Control | Leaves more responsive than soil |
| **TP7–TP8** | Soil | Antimicrobials (Cu, St, Tr) | Significant decrease | Decreased diversity vs Control | Copper & Streptomycin strongest impact |
| **TP9** | Soil | Antimicrobials | No significant difference | Recovery | Temporal resilience observed |
| **TP7** | Leaf | Antimicrobials | Strong sensitivity | **Highest:** Control; **Lowest:** Streptomycin | Phyllosphere most affected |
| **TP8** | Leaf | All | Minor / no differences | ≈ Stable | Effects diminishing |
| **TP9** | Leaf | All | Small residual differences | Slight variation | Near recovery |
| **Community Composition Highlights (TP7–TP9)** | | | |  |  |
| **Time Point** | **Compartment** | **Treatment Group** | **Key Pattern** |  |  |
| **TP7** | Soil | Manure (M) | Enrichment of multiple phyla (Acidobacteria, Proteobacteria, Bacteroidetes) |  |  |
| **TP7** | Soil | GM | Lowest abundance of several dominant taxa |  |  |
| **TP7** | Soil | Control (C) | Highest Actinobacteria & Firmicutes richness |  |  |
| **TP7** | Leaf | Control (C) | Highest overall relative abundance across major taxa |  |  |
| **TP8** | Soil | Manure (M / GM) | Highest diversity of Proteobacteria & Verrucomicrobia |  |  |
| **TP8** | Soil | Control (C) | Distinct proteobacterial enrichment |  |  |
| **TP8** | Leaf | All | No significant compositional difference |  |  |
| **TP9** | Soil | Control (C) | Highest ASV richness overall |  |  |
| **TP9** | Leaf | Glyphosate (G) | Planctomycetes & Proteobacteria enrichment |  |  |
