# Supplementary material for: Dairy manure, glyphosate, and antimicrobials (copper, streptomycin, and triazole) modulated the composition of antimicrobial resistance at the gene and microbial levels in a processing tomato field
Source: Microbiol Spectr. 2026 Mar 17;14(4):e02003-25. doi: 10.1128/spectrum.02003-25 (PMC13055215; doi:10.1128/spectrum.02003-25)
Supplement: Table S5 — Negative correlations in soil samples between the agricultural microbiome and associated antimicrobial resistance gene profiles. [file spectrum.02003-25-s0007.docx]

Supplemental Table 5. Negative correlations in soil samples between the agricultural microbiome and associated antimicrobial resistance gene profiles.

| **Sample type** | **Antimicrobial resistance class** | **Antimicrobial resistance gene** | **ASV (genus/species level)** |
| --- | --- | --- | --- |
| Soil | Aminoglycoside-resistance | aacC1 & 2 and aadA1 | actinobacterium BGR 88 |
|  |  |  | bacterium endosymbiont of Mortierella elongata |
|  |  |  | Bdellovibrionales bacterium Ga0074137 |
|  |  |  | Candidatus Xiphinematobacter americani |
|  |  |  | Chitinophaga sp. WW1(2011) |
|  |  |  | Glomeribacter sp. 1016415 |
|  |  |  | uncultured Chitinophaga sp. |
|  |  |  | uncultured eubacterium WD260 |
|  |  |  | uncultured Flavihumibacter sp. |
|  |  |  | uncultured Holophaga sp. |
|  | Class B beta-lactamase | IMP-2 -5 & -12 | Acidobacteria bacterium 13_2_20CM_56_17 |
|  |  |  | agricultural soil bacterium SC-I-28 |
|  |  |  | agricultural soil bacterium SC-I-71 |
|  |  |  | alpha proteobacterium SK200a-2 |
|  |  |  | bacterium Ellin516 |
|  |  |  | bacterium Ellin6515 |
|  |  |  | bacterium Ellin6543 |
|  |  |  | Candidatus Adlerbacteria bacterium GW2011_GWC1_50_9 |
|  |  |  | endosymbiont of Acanthamoeba sp. KA/E9 |
|  |  |  | Nitrospira japonica |
|  |  |  | Oligoflexus tunisiensis |
|  |  |  | Pennate diatom sp. CCAP 1008/1 |
|  |  |  | planctomycete WY69 |
|  |  |  | Saccharomonospora viridis DSM 43017 |
|  |  |  | uncultured Acidobacterium sp. |
|  |  |  | uncultured bacterium 213 |
|  |  |  | uncultured bacterium 253 |
|  |  |  | uncultured Kofleria sp. |
|  |  |  | uncultured Ktedonobacter sp. |
|  |  |  | uncultured Prosthecobacter sp. |
|  |  |  | uncultured Singulisphaera sp. |
|  |  |  | uncultured sludge bacterium H5 |
|  | Macrolide Lincosamide Streptogramin_b | mefA | agricultural soil bacterium SC-I-81 |
|  |  |  | alpha proteobacterium LWH5 |
|  |  |  | bacterium Ellin5220 |
|  |  |  | bacterium enrichment culture clone auto19_4W |
|  |  |  | delta proteobacterium WX81 |
|  |  |  | Empedobacter sp. PH7-1 |
|  |  |  | Entomoplasma luminosum ATCC 49195 |
|  |  |  | Hymenobacter sp. 9PNM-21 |
|  |  |  | Pontibacter sp. LX8 |
|  |  |  | uncultured Asticcacaulis sp. |
|  |  |  | uncultured bacterium #0319-7E19 |
|  |  |  | uncultured bacterium PHOS-HE28 |
|  |  |  | uncultured Cellvibrio sp. |
|  |  |  | uncultured Dokdonella sp. |
|  |  |  | uncultured Dyadobacter sp. |
|  |  |  | uncultured Ferruginibacter sp. |
|  |  |  | uncultured Thermaerobacter sp. |
|  |  |  | Verrucomicrobia bacterium SCGC AAA027-I19 |
|  | Class C & D beta-lactamase | ACT-1 & MIR and OXA-60 | Actinoplanes sp. 80820 |
|  |  |  | Adhaeribacter terreus |
|  |  |  | Aequorivita echinoideorum |
|  |  |  | Aquabacterium citratiphilum |
|  |  |  | bacterium enrichment culture clone B61(2011) |
|  |  |  | Bdellovibrionales bacterium GWC1_52_8 |
|  |  |  | Berkeleya fennica |
|  |  |  | Caulobacter fusiformis |
|  |  |  | Chitinophagaceae bacterium BLB-2F4-1 |
|  |  |  | Chlamydiae bacterium Ga0074140 |
|  |  |  | delta proteobacterium WY32 |
|  |  |  | Fibrella sp. ES10-3-2-2 |
|  |  |  | Flavobacterium sp. HWG-A1 |
|  |  |  | Frankiales bacterium X5 |
|  |  |  | Frankineae bacterium MT45 |
|  |  |  | Gemmata sp. Br1-2 |
|  |  |  | Gemmatimonadetes bacterium LX87 |
|  |  |  | Gracilibacillus halotolerans |
|  |  |  | Hymenobacter sp. SAFR-023 |
|  |  |  | Luteimonas aestuarii |
|  |  |  | metal-contaminated soil clone K20-27 |
|  |  |  | Minicystis rosea |
|  |  |  | Paenibacillus sp. KAR72 |
|  |  |  | Parcubacteria group bacterium GW2011_GWA1_40_21 |
|  |  |  | Pirellula sp. Br1-4 |
|  |  |  | Prosthecobacter fusiformis |
|  |  |  | Pseudofulvimonas gallinarii |
|  |  |  | Pseudoxanthomonas suwonensis |
|  |  |  | Pythium ultimum |
|  |  |  | Rhizobiales bacterium GB6 |
|  |  |  | Roseomonas sp. SK 65 |
|  |  |  | Sphingoaurantiacus polygranulatus |
|  |  |  | Sphingomonas guangdongensis |
|  |  |  | Sphingomonas sp. Bg12ra |
|  |  |  | Spirosoma sp. 15J9-6 |
|  |  |  | Sporolactobacillus dextrus |
|  |  |  | Stenotrophomonas koreensis |
|  |  |  | Taibaiella sp. |
|  |  |  | Tellurimicrobium multivorans |
|  |  |  | Thermomonas fusca DSM 15424 |
|  |  |  | Thymallus thymallus (grayling) |
|  |  |  | uncultured bacterium 89 |
|  |  |  | uncultured bacterium 92 |
|  |  |  | uncultured bacterium #0319-7F4 |
|  |  |  | uncultured bacterium gp17 |
|  |  |  | uncultured bacterium KF-JG30-B11 |
|  |  |  | uncultured bacterium SBR2013 |
|  |  |  | uncultured Bellilinea sp. |
|  |  |  | uncultured Edaphobacter sp. |
|  |  |  | uncultured Mesorhizobium sp. |
|  |  |  | uncultured Methanomassiliicoccus sp. |
|  |  |  | uncultured Niastella sp. |
|  |  |  | uncultured rumen bacterium 3C0d-16 |
|  |  |  | Vaucheria litorea |

This table only displays negative correlations (P<0.01) between the designated microbiome species and an antimicrobial resistance genes.
